# Editorial: Molecular mechanism of polarized transport in cell polarity

**DOI:** 10.3389/fcell.2025.1616727

**Published:** 2025-08-12

**Authors:** Akihiro Harada, Akiko Kono Satoh

**Affiliations:** ^1^ Department of Cell Biology, Graduate School of Medicine, The University of Osaka, Suita, Japan; ^2^ Program of Life and Environmental Science, Graduate School of Integral Science for Life, Hiroshima University, Hiroshima, Japan

**Keywords:** cell polarity, intracellular transport, Golgi, recycling endosome, TGN, rab, SNARE

Cell polarity has been known to be essential for development and function of various cells and tissues. Cell polarity is established by intracellular traffic of proteins and lipids, known as polarized transport. The molecular mechanisms and the physiological function of polarized transport still remain elusive. Here, we collected a variety of reviews, minireviews, and original articles describing recent advances in the research of polarized transport as listed below. We greatly appreciate the authors who contributed articles to this Research Topic and hope the articles will help those who are interested in this field.

Kunii et al. contribute to this Research Topic with a comprehensive review, “*Molecular mechanisms of polarized transport to the apical plasma membrane*”, summarized the progress in the field of apical transport mainly based on our own results. Polarized transport plays a crucial role in cell polarity. In this review, they introduced the molecular mechanisms of apical transport in particular, and its physiological significance.

In “*Two roads diverged in a cell: Insights from differential exosome regulation in polarized cells*”, Komori et al. reviewed the evidence on biogenesis and exocytosis of exosomes in polarized cells. Exosomes are extracellular vesicles important for intercellular signaling, containing various cargos from miRNAs to proteins. They are released by basically all cells and are extremely heterogenous in their origin and content. Recent advances have revealed specific regulatory pathways for secreting a number of types of exosomes from apical and basolateral sides of polarized epithelial cells. Here they review latest evidence on biogenesis and secretion of exosomes in polarized cells, clarified the challenges that need to be solved, and discuss potential applications of exosomes generated from polarized cells.

In the Mini Review, “*transport mechanisms between the endocytic, recycling, and biosynthetic pathways via endosomes and the trans-Golgi network*”, Toshima and Toshima discuss the differences and similarities of Rabs and SNAREs between yeast and other species. In mammalian cells, endosomes can be roughly classified as early/sorting, late, and recycling endosomes, based on their morphology and localization of Rabs and SNAREs, which are key proteins in vesicle trafficking. However, these endosomes do not necessarily represent specific comparable organelles among different species. For example, Rab5 localizes to early endosomes in mammals but is localized to early-to-late endosomes in yeast, and to the pre-vacuolar endosome and the TGN in plants. The authors revisited the endosome system in mammalian cells by comparing them with the ones in yeast and other species to discuss the differences/similarities between them.

Tago et al. performed a comprehensive study of SNAREs in the fly genome by RNAi in the photoreceptors of *Drosophila* in a brief research report, “*Golgi Clustering by the Deficiency of COPI-SNARE in Drosophila Photoreceptors*”. They indicated that depletion of any of the COPI-SNAREs, resulted in the same phenotypes: Golgi stacks gathering on their trans stacks, Golgi cisternae laterally expanded, and a reduced number of stacks. These Golgi stacks reminded them of mammalian Golgi ribbons and Brefeldin A (BFA)-bodies in *Drosophila* S2 cells. As in the previous report, BFA inhibits trans-Golgi network (TGN) fission and separation of the Golgi stack to form a BFA-body, a cluster of Golgi stacks that have a core of recycling endosomes. We found that the inhibiting each of COPI-SNAREs results in clustered Golgi stacks resembling BFA-bodies, implicating that COPI-SNAREs function to separate clustered Golgi stacks. These results further support the idea that the movement of Golgi stacks and the competition of fusion and fission of the TGN decide the level of clustering and ribbon formation of Golgi stacks.

Zeger et al. study the role of Rab proteins in TRPL recycling using the light-dependent TRPL transport in *Drosophila* photoreceptor cells to in “*tsCRISPR based identification of Rab proteins required for the recycling of Drosophila TRPL ion channel*”. TRPL is localized in the membrane of rhabdomere of dark-adapted flies, but, upon light exposure, it is transported out of the rhabdomere to the ER. By subsequent dark adaptation, TRPL is recycled back to the membrane of rhabdomeres again.

To screen for Rab proteins responsible for TRPL recycling, they established a tissue specific CRISPR-mediated deletion of Rab genes in *Drosophila* photoreceptors and determined the localization of an eGFP-tagged TRPL protein. The authors identified severe TRPL recycling defects in various Rab knockout flies. They exhibited that Rab3 and RabX2 play significant roles in TRPL recycling and transport.

Their study reveals specific Rabs are required for different steps of TRPL transport in photoreceptor cells and showed evidence for a unique retrograde pathway of TRPL recycling from the ER through the trans-Golgi.

Polarized transport is crucial for the development of multiple plasma membrane domains. Photoreceptors of *Drosophila* is a good model system for analyzing the mechanisms of polarized transport. Ochi et al. performed a comprehensive screening of the fly genome using knockdown and knockout combined with the CoinFLP system for the identification of SNAREs involved in post-Golgi trafficking in “*Comprehensive study of SNAREs involved in the post-Golgi transport in Drosophila photoreceptors*”. The results indicate that, no SNARE is responsible for transport from the Golgi to a single specific domain of the plasma membrane. However, each SNARE has a preference for certain membrane domains: the loss of some SNAREs results in rhabdomere transport defects, while the loss of the others leads to basolateral transport impairments. These results suggest that SNAREs are not the only molecules to determine their target domains in the plasma membrane. Furthermore, rhodopsin transport to the rhabdomere needs two R-SNAREs, suggesting that multiple SNAREs are contributing in tandem rather than in parallel.

Joseph et al. showed the role of Rab11b in mitochondria in addition to recycling within cells in “*Rab11b is necessary for mitochondrial integrity and function in gut epithelial cells*”. The RAB11 family are regulators of trafficking of membranes and vesicles. In contrast RAB11A, the function of RAB11B remain unknown. They analyzed RAB11A or 11B interactome and proposed a RAB11B is involved in the regulation of mitochondrial functions. Transcriptomic analysis of intestines of Rab11b knockout mouse revealed a change in mitochondrial functional integrity. An assessment by flow cytometry also revealed a mitochondrial functional impairment *in vivo*. Electron microscopic analysis showed a severe defect of mitochondrial membrane in Paneth cells. These genetic and functional data showed RAB11B is essential for structural and functional maintenance of mitochondria for the first time in addition to its function in membrane recycling.

